# Editorial: Zoonotic negative-sense RNA viruses

**DOI:** 10.3389/fvets.2024.1384858

**Published:** 2024-03-01

**Authors:** Sarah J. Edwards, Jasmina M. Luczo

**Affiliations:** ^1^Health and Biosecurity, Australian Centre for Disease Preparedness, Commonwealth Scientific and Industrial Research Organisation, Geelong, VIC, Australia; ^2^Australian Animal Health Laboratory, Australian Centre for Disease Preparedness, Commonwealth Scientific and Industrial Research Organisation, Geelong, VIC, Australia

**Keywords:** negative-sense RNA viruses, zoonotic viruses, one health, pathogenesis of infection, host pathogen interactions, host response to infection

Zoonotic negative-sense RNA viruses pose a major threat to animal and human health and have caused numerous significant outbreaks, including the 1918 Spanish influenza pandemic (1), the 2009 swine influenza pandemic (2), Ebola virus outbreaks (3), Rift Valley fever virus outbreaks (4), and the H5 highly pathogenic avian influenza virus (HPAIV) panzootic (5). The World Health Organization list of priority pathogens includes numerous negative-sense viruses that pose a significant public health threat due to their epidemic potential and lack of medical countermeasures. As part of outbreak preparedness frameworks, it is crucial to understand factors that contribute to the emergence, maintenance, infection, and spill-over of zoonotic negative-sense viruses.

The aim of this Research Topic was to provide novel insights into zoonotic negative-sense RNA virus biology, disease pathogenesis, host response to infection and countermeasures to mitigate the impact of these viruses.

Currently, H5 goose/Guangdong (gs/Gd)-lineage subclade 2.3.4.4b HPAIVs are causing widespread outbreaks globally. Concerningly, mammal-to-mammal transmission was reported in late 2022 (6). A well-known mammalian adaption of avian influenza viruses (AIVs) is the polymerase basic 2 E627K which enables AIV polymerases to use human ANP32 proteins to replicate (7–9). Briggs and Kapczynski performed a comparative analysis of PB2 E627K/V in H5 AIVs and described low prevalence of E627K in non-gs/Gd-lineages (American, 0.25%; Eurasian 1.03%) and a higher prevalence in the gs/Gd-lineage (range: 0.0–11.7%). In the evolutionary successful H5 gs/Gd-lineage HPAIV subclade 2.3.4.4b, E627K prevalence is 1.0%, suggesting the majority of subclade 2.3.4.4b H5 HPAIVs detected remain adapted to replication in avian species. Notably, E627K was present in 39.1% of human origin H5 AIV sequences, suggesting a non-exclusive requirement.

Vaccination of poultry is considered crucial to protect both animal and public health (10). Park et al. characterized the immunogenicity of a chimeric virus-like particle (VLP) vaccine co-expressing haemagglutinin from gs/Gd-lineage H5 clade 1 and clade 2 (subclade 2.3.2.1c) HPAIVs. The chimeric VLP vaccine elicited a broader antibody response compared to the monovalent VLP vaccine in chickens and ducks. Overall, the VLP vaccine elicited higher and broader serum HI responses in chickens compared to ducks. This VLP vaccine platform enables differentiating infected from vaccinated individuals, making it a valuable tool for the eradication of AIV in poultry whilst ensuring food security. Importantly, vaccination of older ducks will be crucial for HPAIV control, as Lee et al. demonstrated that, whilst young ducks are highly susceptible to severe HPAIV disease and shed higher titres of virus, older ducks do not display clinical disease signs or mortality despite shedding high titres of virus. This highlights the potential for older ducks to maintain and spread HPAIV in the absence of clinical disease signs and suggests that vaccination programs should be targeted toward older ducks.

Another critical area of pandemic preparedness is the development of H5 vaccines for public health. Nuñez et al. demonstrated that immune imprinting with group 1 influenza A viruses (IAVs) (H1N1, H2N3), but not group 2 (H3N2), elicited complete protection following challenge with group 1 H5N1 HPAIV. H3 (group 2) infection of H1 (group 1)-imprinted ferrets did not abrogate protection from H5 HPAIV challenge. Conversely, H5 Hu-COBRA 2 VLP or H5 recombinant HA vaccination of H3 imprinted ferrets afforded protection against H5 HPAIV challenge. Group 1 pdmH1N1 recombinant HA vaccination of H3 pre-immune ferrets elicited partial protection against H5 HPAIV challenge. This study describes group 1 IAVs eliciting cross-reactive protection against heterologous H5 HPAIV challenge and can inform public health vaccination strategies. Complementary to this, the review by Jang et al. of oral mucosal immunity discusses the importance of eliciting sterilizing immunity at the oral mucosa to control transmission of respiratory viruses of pandemic concern. Strategies to improve the induction of sterilizing oral mucosal immunity, including novel mucosal vaccines and adjuvants and delivery systems, were reviewed with current barriers and opportunities described.

Avian influenza viruses are maintained in wild birds and continue to evolve and spread globally. Kim et al. described the detection of Eurasian H6 viruses in South Korea that harbor North American-lineage internal genes, highlighting continual intercontinental spread of AIVs and the need for continued surveillance. The wild bird origin H6 viruses displayed minimal infectivity in chickens, suggesting they were poorly adapted to chickens. Interestingly, Ferreri et al. demonstrated that wild bird-origin H4N2 AIV minimally passaged in chickens acquired increased fitness as evidenced by wider tissue tropism and longer duration of shedding in experimentally infected chickens. This highlights the continued evolution and adaptation potential of AIVs.

Swine IAVs (sIAVs) pose a significant threat to human and swine populations globally. Notably, the pdmH1N1 sIAV emerged in swine from a reassortment event between a triple reassortant sIAV (harboring gene segments from human IAV, sIAV, and AIV) and a Eurasian avian-like sIAV (2). Zeller et al. developed classLog, a general-purpose machine learning classifier to assign taxonomic classifications to virus sequence data without the need to infer evolutionary history. sIAV H1N1 haemagglutinin and Porcine Reproductive and Respiratory Syndrome virus ORF5 datasets were used to validate the classifiers. A classLog classifier trained on a sIAV dataset with 12 features (0.5% of features) and 0% sequence degradation (perfect sequence quality) was 100% accurate. At 10% sequence degradation, 121 features (5% of features) were needed to achieve 100% accuracy. At 20 and 30% sequence degradation, 243 features (10% of features) resulted in 100 and 93% accuracy, respectively. Uncoupling of inference of viral evolutionary history and virus classification increases the rapidity of classification with high accuracy. Importantly, this pipeline can be applied to real time genomic classification in the field. Neasham et al. developed an immortalized swine bronchial respiratory cell line for characterization and risk assessment of sIAVs in a relevant host cell line. The immortalized respiratory cell line was primarily of epithelial origin and maintained epithelial morphology, expressed a-2,3- and a-2,6-linked sialic acid receptors, was permissible to sIAVs and human IAVs, and was functionally immunocompetent, as evidenced by cytokine production.

Finally, Xu et al. undertook a review of Rift Valley fever phlebovirus (RVFV) with a focus on natural hosts and the pathogenesis of RVFV in animal models of infection.

Collectively, this Research Topic highlighted current research activities on zoonotic negative-sense RNA viruses. It is critical to understand viral evolution, infection dynamics, and the host response to infection to inform pandemic preparedness frameworks and develop effective diagnostic tools and countermeasures to combat these viruses.

## Author contributions

SE: Writing—review & editing, Conceptualization. JL: Writing—original draft, Writing—review & editing, Conceptualization.
